# Skin-Expressing lncRNAs in Inflammatory Responses

**DOI:** 10.3389/fgene.2022.835740

**Published:** 2022-04-26

**Authors:** Alanna Shefler, Matthew T. Patrick, Rachael Wasikowski, Jiahan Chen, Mrinal K. Sarkar, Johann E. Gudjonsson, Lam C. Tsoi

**Affiliations:** ^1^ Department of Dermatology, University of Michigan Medical School, Ann Arbor, MI, United States; ^2^ Department of Biostatistics, University of Michigan, Ann Arbor, MI, United States; ^3^ Department of Computational Medicine and Bioinformatics, University of Michigan, Ann Arbor, MI, United States

**Keywords:** lncRNA, atopic dermatitis, psoriasis, keratinocyte, scRNA sequencing

## Abstract

Long non-coding RNAs (lncRNAs) have attracted attention for their potential roles in modulating keratinocyte differentiation and inflammatory response; however, for many identified skin-expressing lncRNAs, there is no comprehensive characterization regarding their biological roles. In addition, the reported expression profiles for lncRNAs can be ambiguous due to their low-expressing nature. The objective of this review is to utilize large scale genomic data to characterize the prominent skin-expressing lncRNAs, aiming to provide additional insights for their potential roles in the pathology of inflammatory skin of psoriasis and atopic dermatitis by integrating *in vitro* and *in vivo* data. We highlighted the different skin-expressing lncRNAs, including *H19*, which is significantly down-regulated in lesional skin of AD/psoriasis and upon cytokine stimulation in keratinocytes; it is also negatively correlated with *CYP1A1* (*r* = -0.75, *p* = 8 × 10^−73^), a gene involved in drug metabolism and skin barrier homeostasis, in keratinocytes. In addition, *SPRR2C*, a potential regulator that modulates IL-22 stimulation, was upregulated in both atopic dermatitis and psoriasis lesional skin and was also downstream of the IL-17A and IL-17 + TNF signaling in keratinocytes. Using scRNAseq, we further revealed the cell type specificity of lncRNAs, including basal-expressing nature of *H19* in the epidermis. Interestingly, instead of having cell type specific expression profile, we found few lncRNAs that are express across different cell types in skin, including *MALAT1*, *NEAT1*, and *GAS5*. While lncRNAs in general have lower expression, our results combining *in vitro* and *in vivo* experimental data demonstrate how some of these lncRNAs can play mediator roles in the cytokine-stimulated pathway.

## Review

Long non-coding RNA (lncRNA) is a class of RNA that does not have protein-coding ability and exhibits its functions as RNA (Derrien et al., 2012; Duan et al., 2020). LncRNAs are >200 nucleotides in length and are commonly located in the chromatin and nucleus (Derrien et al., 2012; Hombach and Kretz, 2013). With the advent of next generation sequencing technologies that enable the detection of low-expressing transcripts, a substantial number of novel lncRNAs have been unraveled. According to the GENCODE consortium (Derrien et al., 2012; Harrow et al., 2012), there were approximately 9,640 lncRNAs in 2010 in human (version 7). In the latest version (v38) released in 2021, the number of lncRNAs approximately doubled with a report of 17,944 lncRNAs (Frankish et al., 2021). NONCODE, a comprehensive lncRNA resource for plants and animals, has reported >170,000 transcripts from 96,000 lncRNAs (Fang et al., 2018). Different studies have revolutionized our understanding of lncRNAs as exhibiting significant regulatory roles rather than being transcriptional waste products (Hombach and Kretz, 2013), and have demonstrated that lncRNAs exhibit stronger tissue-specific expression patterns compared to protein-coding genes (Derrien et al., 2012). LncRNAs have attracted attention due to their role in gene regulatory processes, maintaining normal tissue homeostasis, and transition to diseased states (Hombach and Kretz, 2013; Tsoi et al., 2015). They have been shown to modulate epigenetic regulation of chromatin (Rinn et al., 2007; Gupta et al., 2010; Tsai et al., 2010; Heo and Sung, 2011; Wang et al., 2011; Grote et al., 2013; Hombach and Kretz, 2013; Tsoi et al., 2015), as well as participating in promoter-specific gene regulation (Hombach and Kretz, 2013; Tsoi et al., 2015; Duan et al., 2020), X-chromosome inactivation (Tian et al., 2010; Hombach and Kretz, 2013; Tsoi et al., 2015; Duan et al., 2020), and imprinting (Lee and Bartolomei, 2013). Therefore, they are associated in various diseased states, such as neurodegenerative conditions (Wapinski and Chang, 2011), susceptibility to infection (Gomez et al., 2013), and different types cancers (Huarte et al., 2010; Prensner et al., 2011; Maruyama and Suzuki, 2012).

Skin diseases are the fourth most common cause of illness, whereby up to 33% of the population is affected (Bickers et al., 2006; Hay et al., 2014; Hay et al., 2015; Tizek et al., 2019). Many of these diseases display strong inflammatory components whose mechanisms have not been fully explained. Recent genomic studies highlight how multiple lncRNAs can be implicated in key roles for epidermal homeostasis, transition to disease state, and stress response (Hombach and Kretz, 2013; Tsoi et al., 2015) (Table 1; Supplementary Table S1). Using RNA sequencing, previous work has revealed that *DANCR* (anti-differentiation non-coding RNA) is significantly downregulated during terminal differentiation (Huarte et al., 2010; Kretz et al., 2012), and indicates its functional role in the transition from non-differentiated to differentiated cells (Huarte et al., 2010). *DANCR* may also repress differentiation of osteoblasts and keratinocyte progenitors by associating with methyltransferase EZH2, a component of the chromatin modifying protein complex PRC2, which is involved in epigenetic silencing via histone methylation (Cao et al., 2002; Hombach and Kretz, 2013; Zhu and Xu, 2013). Kretz et al. illustrated that lncRNA *TINCR* (terminal differentiation induced non-coding RNA) is upregulated in epidermal differentiation and forms a complex with STAU1 (staufen double-stranded RNA binding protein), which increases stabilization of differentiation (Kretz et al., 2013). Together, *DANCR* and *TINCR* are essential in maintaining epidermal homeostasis (Hombach and Kretz, 2013; Kretz et al., 2013) in cells under normal state, however their behaviors in inflammatory skin conditions are not well studied. Another study that focused on the roles of lncRNAs in cutaneous biology demonstrated that lncRNA *BC020554* is downregulated while *AK022798* is upregulated during keratinocyte differentiation (Tang et al., 2020). However, for the majority of the skin-expressing lncRNAs, there is limited information regarding their biological and pathological roles in cutaneous disorders.

**TABLE 1:** The fold change for the notable lncRNAs that play roles in skin biology and/or diseases. Bold numbers denote FDR ≤0.05. Red results denote at least 1.5-fold change when comparing against control/unstimulated conditions. Expression in inflammatory skin disease represents findings in lesional skin of atopic dermatitis/psoriasis patients compared to control without skin disease.

lncRNA	Cell type detected	Expression in inflammatory skin disease		Expression in cytokine stimulated keratinocytes							Role
		AD	Pso	IFNa	IFNg	IL4	IL13	IL17A	IL17 + TNFa	TNFa	
*DANCR*	KC	1.16	**1.88**	**0.92**	**0.73**	0.99	0.99	1.02	**0.88**	**0.89**	Helps maintain the non-differentiated state in the epidermal basal layer by associating with EZH2 (Hombach and Kretz, 2013; Tang et al., 2020)
*TINCR*	KC	**0.67**	**0.65**	1.00	**0.68**	**1.09**	**1.07**	0.97	**0.77**	**0.94**	Interacts with differentiation mRNAs and helps them bind to the STAU1 protein, increasing their stabilization (Hombach and Kretz, 2013; Tang et al., 2020)
*H19*	KC	**0.31**	**0.05**	0.85	**0.71**	**0.82**	0.94	0.92	**0.71**	0.89	Promotes keratinocyte differentiation by targeting MiR-130b-3p and inhibiting its activity on Dsg1 (Li et al., 2017; Tang et al., 2020)
*NEAT1*	**Psoriatic Epidermis**	1.14	**1.44**	**0.97**	**0.74**	0.96	0.91	1.02	0.97	**0.93**	Upregulated in AD and in proliferation of hemangioma tissues (Tang et al., 2020)
*RNU1-1*	KC	NA	**1.63**	1.01	1.00	1.00	1.01	1.00	1.01	1.00	Indicator for sun-induced skin injury. Change in U1 RNA structure by UVB induces cytokine expression (Hombach and Kretz, 2013)
*UCA1*	**KC**	**1.76**	**2.97**	**0.60**	**0.68**	**0.71**	0.76	**0.77**	**0.72**	0.71	Upregulates expression of A20 by inhibiting miR125a, which decreases activity of NF κ B. It also downregulates IL-6, IL-8, IFN-y and inhibits melanogenesis (Tang et al., 2020; Ma et al., 2021)
*SPRR2C*	**KC**	**8.51**	**245.06**	1.01	1.23	0.87	0.96	**1.88**	**6.92**	1.14	Important in psoriasis pathogenesis and response to treatment. Upregulated in both psoriatic lesional skin and HaCaT cell lines in response to IL-22 treatment (Luo et al., 2021)
*GAS5*	**Psoriatic Epidermis**	NA	1.06	0.98	**0.83**	0.96	0.97	0.96	**0.92**	**0.90**	Indicator of psoriasis severity. Positive correlation between GAS5 and PASI (Ahmed Shehata et al., 2021)
*MALAT1*	**Psoriatic Epidermis**	1.08	**1.02**	1.02	**0.76**	0.94	1.07	1.03	1.12	0.98	Promote melanoma cell proliferation, invasion, and migration (Tang et al., 2020)

Psoriasis is a chronic, relapsing, hyperproliferative, inflammatory skin disease whereby the epidermis is thickened secondary to abnormal differentiation of basal keratinocytes (Szegedi et al., 2010; Tang et al., 2020). LncRNA *PRINS* (psoriasis susceptibility related RNA gene induced by stress) has been shown to contribute to psoriasis susceptibility and is overexpressed in psoriatic uninvolved skin (Sonkoly et al., 2005; Zenz et al., 2005; Bari et al., 2011; Szegedi et al., 2012; Hombach and Kretz, 2013; Tsoi et al., 2015). It is also involved in cellular stress response and may play a role in psoriasis by decreasing sensitivity to keratinocyte apoptosis via the regulation of G1P3 (Sonkoly et al., 2005; Zenz et al., 2005; Bari et al., 2011; Szegedi et al., 2012). Ma et al. demonstrated that lncRNA *UCA1* (urothelial carcinoma-associated 1) was downregulated in psoriatic lesional skin (Ma et al., 2021). Initially named for its high expression in bladder cancer cells (Roberson and Bowcock, 2010; Ma et al., 2021), *UCA1* has now gained more attention regarding its role in psoriasis (Ma et al., 2021). Specifically, it upregulates the expression of cytoplasmic zinc finger protein A20 (TNFAIP3, TNFα induced protein 3) by inhibiting microRNA125a (Ma et al., 2021). Through the miRNA125a-A20 axis, *UCA1* negatively regulates the activity of NF κ B (Li et al., 2015; Ma et al., 2021). Other lncRNAs that have revealed roles in psoriasis include lncRNA *RP6-65G23.1*, lncRNA *MSX2P* (cytoplasmic lncRNA Msh homeobox 2 pseudogene 1), and lncRNA *GAS5* (growth arrest specific 5). LncRNA *RP6-65G23.1* is upregulated in psoriatic keratinocytes and associated with increased anti-apoptotic cells (i.e., BCL2 and Bcl-xL) and cell proliferation (Lizzul et al., 2005; Duan et al., 2020). LncRNA *MSX2P* is also upregulated in psoriatic lesional skin (Kretz et al., 2013) and involved in IL-22 induced keratinocyte growth. LncRNA *GAS5* is a strong indicator of psoriasis severity (Ahmed Shehata et al., 2021). While these studies have helped to gather biological insight for these newly uncovered lncRNAs, the reported expression profiles are sometimes ambiguous (see below), and there is no comprehensive catalog regarding most skin-expressing lncRNAs’ biological and pathological roles.

Therefore, in this review we aim to characterize the expression profiles for the prominent skin-expressing lncRNAs, aiming to provide additional insights for their potential roles in atopic dermatitis and psoriasis pathology by integrating *in vitro*, *in vivo*, and *in silico* techniques. First, we identified candidate lncRNAs that were previously identified in the literature to play a role in cutaneous biology. We then evaluated the gene expression patterns in *in vivo* lesional atopic dermatitis and psoriasis skin to identify the differentially expressed lncRNAs (Tsoi et al., 2019a; Tsoi et al., 2019b). We evaluated the lncRNA profiles from published transcriptomes in non-lesional and lesional skin from 27 atopic dermatitis and 28 psoriatic patients, as well as from 38 healthy controls. This information was integrated with *in vitro* genomics data of cytokine-stimulated conditions (IFNa, IFNg, IL-4, IL-13, IL-17A, IL-17 + TNFa, TNFa) from 40 patients to infer the regulatory mechanism upon inflammatory response for keratinocytes (Tsoi et al., 2019a; Tsoi et al., 2019b). We also conducted correlation analysis for the *in vivo* and *in vitro* data to associate expression profiles of the candidate lncRNAs with the other prominent genes involved in skin inflammation. To reveal lncRNA-expressing cell type in the *in vivo* data, we assayed the expressions in scRNA-seq data from non-lesional and lesional skin.

### Differentially Expressed lncRNAs in Cytokine Stimulated Keratinocytes and *In-Vivo* Lesional Skin

**FIGURE 1:**
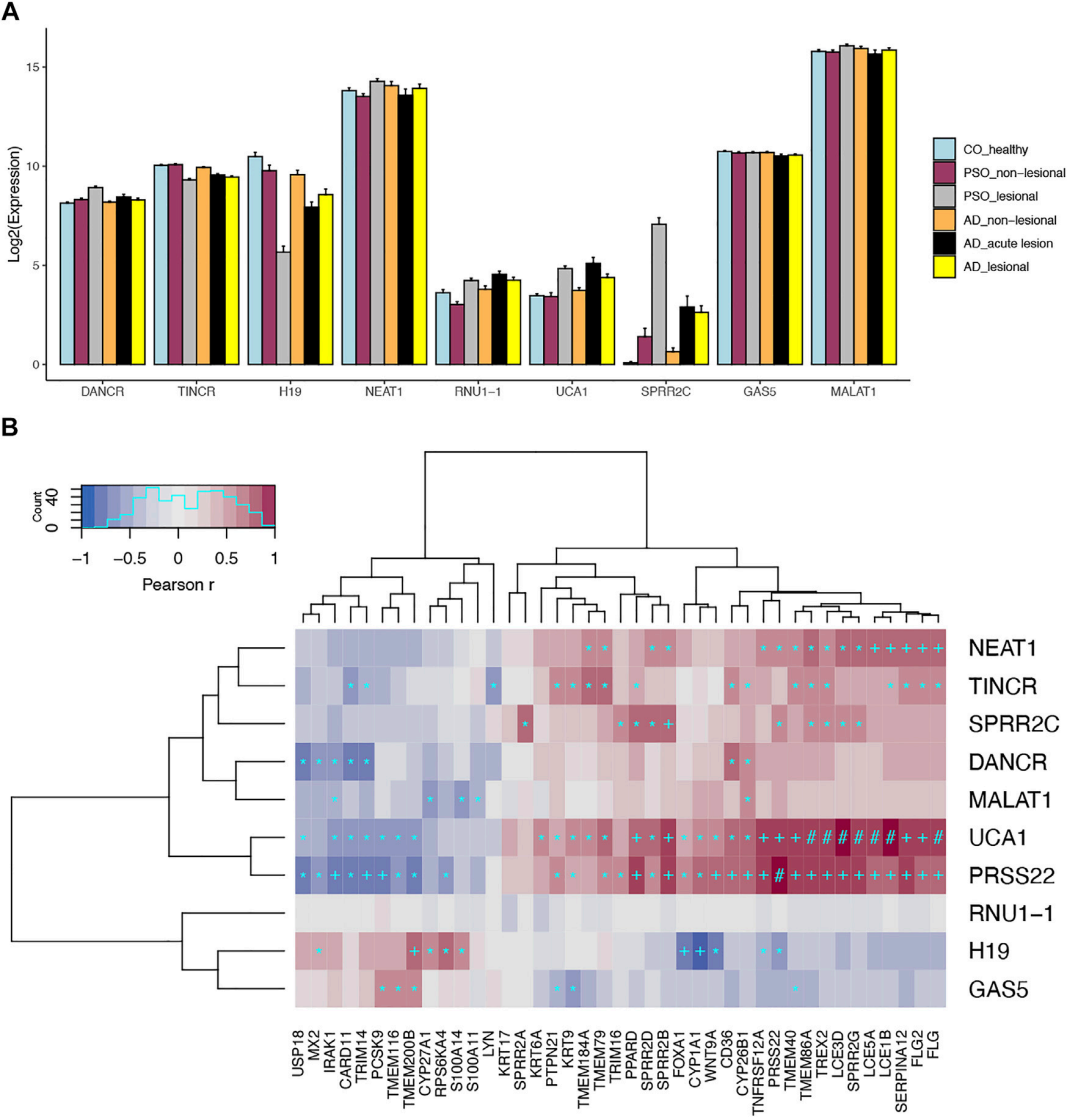
Expression profiles for skin-expressing lncRNAs. **(A)** Gene expression profiles for prominent skin-expressing lncRNAs in different disease conditions. **(B)** Top most correlated protein-coding genes for skin-expressing lncRNAs. Heatmap shows the spearman correlation in keratinocytes. *p < 1x10 ^*−20*^ *;* +*p < 1x10* ^*−50*^ *;* #*p < 1x10* ^*−100*^.

The summary statistics for the above lncRNAs detected in our analysis were presented in Table 1. As previously discussed, *DANCR* and *TINCR* play critical roles in maintaining epidermal homeostasis (Huarte et al., 2010; Kretz et al., 2012). *DANCR* is expressed in non-differentiating keratinocytes while *TINCR* is expressed in differentiating keratinocytes (Piipponen et al., 2020). When evaluating gene expression patterns in lesional compared to control skin, *DANCR* was upregulated and *TINCR* was downregulated in psoriasis lesional skin (Figure 1A). These findings support our current understanding of psoriasis as an inflammatory skin disease that is in part characterized by poor differentiation of keratinocytes (Ma et al., 2021). Of importance, we discovered that *TINCR* was also downregulated in atopic dermatitis lesional skin—a finding that has not previously been reported. Similar to *TINCR*, lncRNA *H19* also regulates keratinocyte differentiation by targeting MiR-130b-3p and inhibiting its activity on Desmoglein 1 (Dsg1) (Li et al., 2017), which is expressed mainly in the superficial layer of the epidermis and mediates cell-cell adhesion. We found that *H19* was downregulated in both atopic dermatitis (Fold Change *FC =0.31; FDR = 3.26x10* ^*−6*^) and psoriasis (Fold Change *FC = 0.05*; *FDR = 3.34x10* ^*−15*^) lesional skin, and it was also depleted in the IFN and IL17/TNF stimulated keratinocytes, further supporting our understanding of aberrant differentiation of keratinocytes in many skin diseases, such as psoriasis and atopic dermatitis (Li et al., 2017; Tang et al., 2020).

Prominent skin-expressing lncRNAs that were upregulated in both psoriasis and atopic dermatitis lesional skin include *UCA1* and *SPRR2C* (small proline rich protein 2C) (Table 1). Through the miRNA125a-A20 axis, *UCA1* has been shown to downregulate NF κ B which plays an important role in linking psoriatic keratinocytes and immune cell states (Ma et al., 2021). However, *UCA1* has previously been reported to be downregulated in psoriatic keratinocytes (Ma et al., 2021) for patients with elevated NF κ B signaling. Our analysis therefore suggests heterogeneity in inflammatory signaling among psoriatic patients, in concordance with our recent findings that show the association between variations in molecular signature and anti-TNF drug response (Tsoi et al., 2021). Indeed, we observed that *UCA1* can be associated with NF κ B signaling (see correlation analysis below). *SPRR2C* has also been shown to play an important role in psoriasis pathogenesis and response to treatment (Luo et al., 2021). Prior studies have indicated that it is upregulated in psoriasis lesion skin (Luo et al., 2021), consistent with our analysis (Table 1; Figure 1A). Moreover, though with a lesser degree than that in lesional skin, *SPRR2C* is significantly upregulated in both the non-lesional skin of psoriasis and atopic dermatitis (Figure 1A), and it is also induced through IL17 and IL17 + TNF stimulation in keratinocytes; while the dysregulation of *UCA1* was also observed in the lesional skin but not the non-lesional skin of AD and psoriasis. While many of the more notable lncRNAs listed in Table 1 do not seem to respond significantly to cytokine stimulation of keratinocytes, previous studies have highlighted a substantial portion (∼40%) of all the >1,000 skin-expressing lncRNAs are dysregulated in the lesional skin of psoriasis and atopic dermatitis (Tsoi et al., 2015; Tsoi et al., 2019b), suggesting that we still have very limited knowledge in understanding potential biological regulation and roles for this type of transcript.

## Co-Expression Analysis Revealed Potential Biological Modules

Our previous work utilized co-expression patterns to infer potential biological roles of lncRNAs (Tsoi et al., 2015). In this study, we also found significant correlations between the aforementioned lncRNAs with other genes known to play roles in skin diseases (Figure 1B). For instances, we observed strong negative correlation (*r* = −0.75, *p* = 8 × 10^−73^) between *H19* and *CYP1A1* in keratinocytes. In addition to its important impact on drug metabolism (Mescher and Haarmann-Stemmann, 2018), *CYP1A1* is involved in the AHR pathway, which controls skin barrier homeostasis (Kyoreva et al., 2021). Meanwhile, *SPRR2C* is positively correlated with other genes encoding for small proline-rich proteins (i.e., *SPRR2D*, *SPRR2B*, *SPRR2G*), which are structural components of the cornified epithelia; and it is also correlated with *IL36A* (*r* = 0.55, *p* = 8 × 10^−33^), *IL36G* (*r* = 0.58, *p* = 5 × 10^−37^) and *IL36RN* (*r* = 0.57, *p* = 5 × 10^−36^), a group of cytokines which are involved in skin inflammation (not shown in Figure 1B) (Hernández-Santana et al., 2020) and contribute to the pathogenesis of psoriasis and atopic dermatitis (Tsang et al., 2020). Notably, IL-36 signaling has been shown to induce IL-17 and IL-23 signaling (Goldstein et al., 2020), which corresponds with our finding that *SPRR2C* is up-regulated upon IL-17 stimulation. Other interesting correlations we discovered include *UCA1* with filaggrin genes, *FLG* (*r* = 0.84, *p* = 4 × 10^−106^), *FLG2* (*r* = 0.82, *p* = 3 × 10^−99^), as well as late cornified envelope genes, *LCE1B* (*r* = 0.89, *p* = 7 × 10^−141^), *LCE3D* (*r* = 0.87, *p* = 4 × 10^−127^), *LCE3E* (*r* = 0.85, *p* = 1 × 10^−110^), *LCE5A* (*r* = 0.85, *p* = 7 × 10^−112^). Filaggrin deficiencies are an important risk factor for atopic dermatitis, as they weaken the epidermal barrier (Weidinger et al., 2018). LCE genes, which are associated with psoriasis, have been found to have antibacterial activity (Niehues et al., 2017). Of note, *UCA1* was also significantly correlated with *TNFSF12A* (*r* = 0.75, *p* = 2 × 10^−73^) in keratinocytes, which is an important regulator for the NF κ B signaling pathway (Xia et al., 2021).

### lncRNA as Cell Type Signature

**FIGURE 2:**
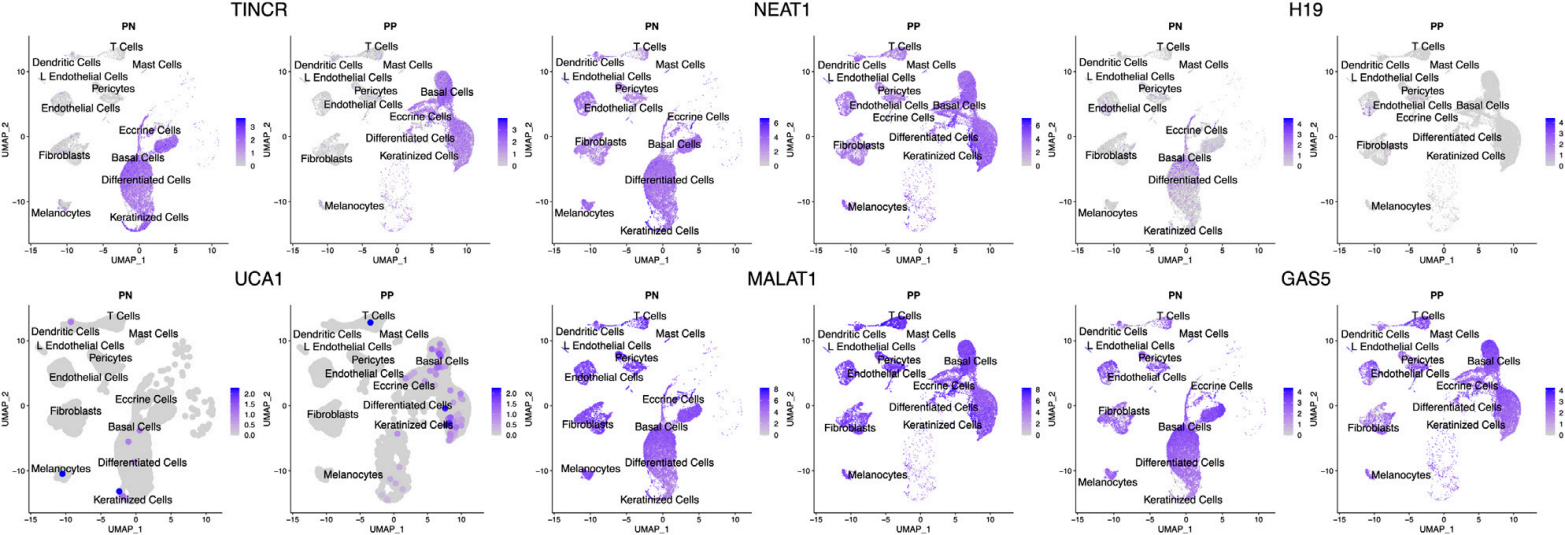
Expression of lncRNAs across different cell types. UMAP of the scRNA-seq data highlights the cell type specific/shared expression profiles for different lncRNAs. The intensity of blue corresponds to the expression level of the lncRNAs in each cell. The cell type annotations label the cell clusters nearby. PP denotes lesional psoriasis skin, while PN denotes nonlesional psoriasis skin.

We looked up the expression architecture for these lncRNAs in the psoriatic and non-lesional skin scRNA-seq data. Interestingly, despite the well-known cell type specific nature of lncRNAs, we observed that some highly expressed lncRNAs, such as *NEAT1*, *MALAT1*, and *GAS5* are expressed in all cell types in skin (Figure 2). Others, including *UCA1*, *H19*, and *TINCR*, are expressed only in keratinocytes. However, we were not able to detect *SPRR2C*, likely due to the lack of sensitivity in detecting low-expressing transcripts for lncRNAs. These lncRNA expression patterns allow us to better understand the cell types that these lncRNAs could exert their functionality. Notably, we observed *H19* expression mostly in the basal layer of the epidermis, and the scRNA-seq data further confirmed that it is down-regulated in lesional skin. In comparison, *TINCR* was found to be expressed in all of the compartments in the epidermal layer. We also replicated the higher expression of *UCA1* in the lesional skin of psoriasis, and its expression seems to appear throughout the epidermis while slightly higher in the keratinized layer.

## Summary

With the advent of next generation sequencing technology, our understanding and characterization of lncRNAs has accelerated (Ponting et al., 2009). As a class, lncRNAs are preferentially enriched in the nucleus, do not have coding protein ability, and exhibit more tissue-specific expression patterns compared to protein-coding genes (Derrien et al., 2012). Over recent years, they have attracted attention due to their roles in epidermal homeostasis, transition to disease states, and stress response (Hombach and Kretz, 2013; Tsoi et al., 2015). In this review, we characterized the expression profiles for the prominent skin-expressing lncRNAs and provided additional insights for their potential roles in atopic dermatitis and psoriasis pathology by integrating *in vitro*, *in vivo*, and *in silico* techniques. We were able to better understand the behaviors for some of the reported skin-specific lncRNAs, as well as their architecture, by integrating single cell RNA-sequencing.

While *TINCR* is known to play a role in keratinocyte differentiation (Kretz et al., 2013), its dysregulation in atopic dermatitis and psoriasis has not yet been fully understood. Although its downregulation supports our understanding of aberrant differentiation in these diseases (Li et al., 2017), its role in modulating skin differentiation under inflammatory environment require further investigation. Our results also highlight that some of the prominent skin-expressing lncRNAs can be found in multiple different cell types, while others exhibit cell type specific expression patterns. These findings can help us to better understand the cell type specific/shared regulation programming. A higher throughput or transcript-targeted single cell transcriptomic approach can provide a better resolution in revealing expression patterns for more lncRNAs, especially for those that are low-expressing.

In conclusion, this review provided additional insights for the skin-expressing lncRNAs that contribute to atopic dermatitis and psoriasis. It further helped facilitate our understanding of the role of lncRNAs upon cytokine stimulation and how this can relate to autoimmune diseases. We discovered shared and unique expression, as well as cell type specificity, for lncRNAs in non-lesional and lesional skin of psoriatic patients. This analysis should provide valuable information for future studies that target lncRNAs for biomarker development and pharmaceutical intervention.

## Supplementary Material

Supplementary Table S1 Other previously identified lncRNAs that play roles in skin biology and/or diseases.
