# Supplementary material for: Skin-Expressing lncRNAs in Inflammatory Responses
Source: Front Genet. 2022 Apr 26;13:835740. doi: 10.3389/fgene.2022.835740 (PMC9086234; doi:10.3389/fgene.2022.835740)
Supplement: Supplementary file 1 [file Table1.pdf]

Supplementary Table 1. Other previously identified lncRNAs that play roles in skin biology and/or diseases.

lncRNA	Cell Type Detected	Role
BC020554	KC	Downregulated during differentiation. Exact role unknown.
AK022798	KC	Upregulated during differentiation. Exact role unknown.
WAKMAR1	KC	DNMT-associated lncRNA that increases keratinocyte motility and re-epithelialization, which is important in wound healing [55].
PRINS	Psoriatic Epidermis	Overexpressed in epidermis of uninvolved psoriatic skin [29]. Plays a role in psoriatic susceptibility, cellular stress response, and regulation of apoptosis.
RP6-65G23.1	Psoriatic Epidermis	Upregulated in psoriatic keratinocytes and is associated with increased anti-apoptotic cells (ie BCL2, bcl-xl). Can activate ERK1/2 and AKT pathway leading to G1/S progression, thereby increasing cell proliferation [2, 38].
MSX2P1	Psoriatic lesions	Upregulated in psoriatic lesional skin. Important in assisting in the progression and growth of IL-22 stimulated keratinocyte by binding miR-6731-5p and activating S100A7 [28].
